# Retraction of: Mitochondrial Genome Sequencing with Short Overlapping Amplicons on Miseq FGx System

**DOI:** 10.1093/fsr/owae031

**Published:** 2024-05-15

This is a retraction of: Yang Xin, Rulin Jia, Suhua Zhang, Fei Guo, Mitochondrial Genome Sequencing with Short Overlapping Amplicons on Miseq FGx System, *Forensic Sciences Research*, Volume 7, Issue 2, June 2022, Pages 142–153, https://doi.org/10.1080/20961790.2021.1963514

In December 2021, *Forensic Sciences Research* published the article “Mitochondrial Genome Sequencing with Short Overlapping Amplicons on Miseq FGx System” by Yang Xin, Rulin Jia, Suhua Zhang, and Fei Guo. https://doi.org/10.1080/20961790.2021.1963514

In January 2024, the publisher was alerted to concerns regarding the applicability of the ethical approval for the research, the validity of the informed consent to participate in the research published in the article, and the validity of the informed consent to publish individual data presented in supplementary files to the article. The authors have not provided documentation demonstrating that they received sufficient ethical approval from a local ethical committee for the research, and as such the Editors-in-Chief and the Publisher have taken the decision to retract this paper. The authors have been informed of this decision and do not agree with the retraction. The supplementary files to the article have been removed given doubts over the validity of the informed consent to publish individual data.

